# Influence of Nutritional Education on the Diet and Nutritional Behaviors of Elderly Women at the University of the Third Age

**DOI:** 10.3390/ijerph17030696

**Published:** 2020-01-21

**Authors:** Małgorzata Magdalena Michalczyk, Izabela Zajac-Gawlak, Adam Zając, Jana Pelclová, Robert Roczniok, Józef Langfort

**Affiliations:** 1 Institute of Sport Science, The Jerzy Kukuczka Academy of Physical Education, Mikolowska 72a, 40-065 Katowice, Poland; i.zajac-gawlak@awf.katowice.pl (I.Z.-G.); a.zajac@awf.katowice.pl (A.Z.); r.roczniok@awf.katowice.pl (R.R.); langfort@imdik.pan.pl (J.L.); 2 Faculty of Physical Culture, Palacký University Olomouc, Tr.Míru 115, 77 111 Olomouc, Czech Republic; jana.pelclova@upol.cz

**Keywords:** elderly, diet, VFA, physical activity, healthy lifestyle

## Abstract

Background: The objective of this study was to evaluate the diet composition, body fat content, and physical activity (PA), considering blood lipid levels and insulin resistance markers, in elderly women who were well educated in nutrition and healthy lifestyle choices. Methods: A total of 106 postmenopausal women took part in the study. The study group included 62 students from the University of the Third Age (U3A); the control group (CG) included 44 females from the Silesia region. We evaluated their daily macro and micronutrient intake, levels of PA, percent of body fat (PBF), and the visceral fatty area (VFA). We also evaluated the lipid profile, insulin and glucose levels, homeostatic model assessment of insulin resistance (HOMA-_IR_), and C-reactive protein (CRP) levels. Results: Significant differences were observed in carbohydrate, protein, fiber, as well as vitamins and minerals consumption between the U3A group and the CG. There were no differences in the PBF and VFA between the groups. Furthermore, no differences were shown in the measured blood variables. The U3A group walked more than 11,000 steps a day and performed 46.15 min/day of PA with a moderate intensity of 3–6 metabolic equivalents of task (METs, min/week). Conclusions: Despite the fact that the U3A group were physically active females, well educated on healthy, balanced diets and had the motivation to learn about proper nutritional behaviors, they did not follow these recommendations in everyday life.

## 1. Introduction

Older adults are the most rapidly growing age group in Western countries [1,2,3]. Unfortunately, most of them suffer from chronic diseases and changes in their lean body mass and fat mass [4,5], which are often due to incorrect dietary habits. The lack of adequate quality and quantity of nutrients, even of a macronutrient like fat, contributes to negative changes in their body composition [1]. Excess carbohydrate and fat consumption, in particular, promotes the development of obesity and diabetes [6].

Aging is accompanied by a variety of physiological, psychological, economic, and social changes that may adversely affect nutritional status [4,7,8]. The consumption of a Western-style diet, rich in fats and sugar, in the elderly population enhances the risk of obesity, diabetes mellitus, atherosclerosis, and other diseases [1,2,4,9,10]. Therefore, it becomes crucial to educate the elderly about proper nutrition in order for them to understand how a healthy, balanced diet influences general health and aging [8].

A balanced diet should include an appropriate proportion of each macronutrient and adequate caloric content, adapted to age, sex, and lifestyle [6,11,12]. As people age, they undergo physiological changes that are reflected by decreases in total energy intake, compared with what is recommended, while consuming inappropriate amounts of macronutrients [4,5,6,13,14,15,16]. A lower calorie intake has been associated with lower consumption of protein, calcium, iron, zinc, B vitamins, and vitamin E and D, which can advance the aging process and other diseases [4,8,17,18,19]. Many factors, such as education, socioeconomic factors, and marital status, may influence a decline in energy intake [6,13]. Recent epidemiological studies indicate that changes in the diets of older women result from dissatisfaction with their bodies; there is evidence that this phenomenon is similar in girls and young women [6,20,21]. Partially, physiological changes in the digestive track (e.g., slower gastric emptying, slower intestinal peristalsis, and altered taste and smell) may also contribute to lower energy intake [10,21,22]. 

The aging process has a crucial impact on decreasing the lean body mass and increasing body fat in the visceral fatty area (VFA) [5,10,11,21,23]. Apart from a balanced diet that includes appropriate protein consumption, daily physical activity (PA) is important to maintain a proper VFA [10,21,22]. Regular PA at moderate intensity facilitates weight loss and induces favorable changes in body composition, thus reducing the risk of becoming overweight and developing obesity [3,16,23]. PA also accelerates intestinal peristalsis and reduces the development of other diseases [3,4,11,23]. According to the World Health Organization, performing 10,000 steps/day at a moderate intensity is considered a minimum standard for general health [10,24,25]. Furthermore, the American College of Sports Medicine indicates that 150 min a week of moderate-intensity PA (3–6 metabolic equivalents of task (METs)) can reduce the risk of several diseases, while 300 min a week will help improve overall health and fitness [26].

A healthy diet is an important factor in healthy aging [3,14,26]. Pro-health nutritional education may significantly improve people’s health-related quality of life [12,27,28]. Therefore, in the current study, we assessed the nutrient and energy consumption, body composition, levels of PA, blood lipid variables, and insulin resistance biomarkers in women over 60 years old.

## 2. Material and Methods

### 2.1. Study Sample

A total of 106 postmenopausal women volunteered to participate in the study. The anthropometric characteristics of the subjects are given in Table 1. The study group included 62 female students from the University of the Third Age (U3A), located in the upper Silesia region of Poland. The control group (CG) included 44 females with no education in the area of nutrition; they were residents of the Silesia region. In the U3A group (n = 62), 52 women were married, 5 were divorced, 2 were single, and 2 were widows. In the CG (n = 44), 39 were married, 4 were divorced, and 1 was single. In the U3A group, 42% had higher education, 32% had secondary education, and 26% had vocational education. In the CG, 45% had higher education, 37% had secondary education, and 18 had vocational education. The inclusion criteria for the study group were as follows: low levels of PA before the study, no chronic diseases, and no intake of medications that may affect the serum lipid profile or the glucose level. Subjects were informed of the purpose and possible risks of the investigation. Written informed consent was obtained from all participants. The study protocol was approved by the local Ethics Committee at the Academy of Physical Education in Katowice, Poland (ethic references KB- 03/2017). 

### 2.2. Diet Analysis

Participants were asked to prepare a 1 week (five week days and two weekend days) dietary record to assess their habitual daily energy and nutrient consumption. To prepare the dietary record, they were asked to weigh all consumed products and record the volume of fluids they drank during the day [29]. While preparing a meal, subjects used kitchen scales to precisely measure the amount of each product consumed. To determine the dietary nutritional composition, the National Food and Nutrition Institute database—Dieta 5D (National Food and Nutrition Institute, Poland) was used. 

### 2.3. PA Measurement

The PA level was assessed based on the number of steps taken per day, using 10,000 steps/day as a criterion to classify individuals as “active” [26]. The PA level was also assessed by considering the minutes of moderate-intensity PA, reflected as 3–6 METs (kJ/kg/h) per day (min/d). The MET was used to measure exercise intensity; one MET corresponds to 3.5 mL O_2_/kg/min oxygen uptake at rest and it is 4184 kJ/kg/h. The measurement of PA was conducted over 14 h/day, on average, for seven consecutive days, using Actigraph GT1M. This fulfilled the accelerometer wear-time required to obtain a reliable estimate of PA in older adults [30]. Participants placed the Actigraph in the small pocket of the elastic belt that they wore positioned near their right iliac crest. The belts were only removed during water exercises and before going to bed. To minimize the potential influence of participant’s reactivity and to be sure that PA measurements were more objective, participants wore the Actigraph for 8 days (≥10 h of wear time a day), although the first day’s readings were not considered in the analysis [31]. The time sampling interval was set at 60 s epochs. In this study, the accelerometer output in counts per minute (cpm) was derived using adult population cut-offs. The intensity ≥1952 cpm corresponded to moderate-to-vigorous PA [31]. Non-wear time was defined as ≥60 consecutive minutes of zero activity counts, allowing interruptions of counts above 0 for two minutes. In order to establish the Activity Factor (AF) of participants from the CG, a validated International Physical Activity Questionnaire (IPAQ) was filled out. 

### 2.4. Body Mass and Composition Evaluation 

After an overnight fast and a minimum of 3 days without alcohol consumption, each participant reported to the laboratory in the morning for body mass evaluation and blood analyses. The evaluation of body composition, basal metabolic rate (BMR,) and adipose tissue content was performed by multifrequency bioimpedance analysis (MF-BIA) using the InBody 720 (Biospace Co., Ltd., Seoul, Korea). MF-BIA is a useful and convenient substitute for computed tomography in measuring VFA (cm^2^) [32]. The body mass index (BMI) was calculated using the following formula (BMI = body mass/height = kg/m^2^). The total daily energy expenditure (TDEE) was calculated according to the commonly accepted model (TDEE = BMR × AF) [29]. The AF was determined based on the available indicator (high activity: 2.0; medium: 1.6; low: 1.4; and sedentary lifestyle: 1.2) [29]. Among body composition components, we determined the VFA, which was defined as a cross sectional area of visceral fat in the abdomen at the umbilical level (L_4_–L_5_). An upper limit of 100 cm^2^ was accepted for the VFA. Waist circumference (WC) was also measured. WC measurements were taken with an anthropometric tape (to the nearest 0.5 cm) at midway between the lowest rib and the iliac crest of the standing participant. The measurements were taken under laboratory conditions, according to the instructions of the manufacturer. 

### 2.5. Biochemical Analysis

Fasting blood samples were collected in the morning (around 8:00 a.m.), after body mass analysis. Vacutainer tubes were used to determine the glucose, insulin levels, and lipid profile. Blood serum was separated using routine procedures and either processed immediately or kept frozen at −70 °C until analysis. The concentration of the total plasma cholesterol (tCh), high-density lipoprotein cholesterol (HDL-C), and triacylglycerol (TG) was enzymatically determined in duplicate using commercially available kits from Randox Lab (CH 201-tCh, TR 210-TG, CH 203-HDL-C, respectively). Concentration of low-density cholesterol (LDL-C) was calculated from tCh, HDL-C, and TG using the Friedewald equation [33]. The plasma glucose concentration was determined using the diagnostic kit GL (2623) from Randox Lab. Serum insulin (I) was assessed by an immunoradiometric assay using the IRMA kit (IM3210; Czech-Republic). The HOMA-_IR_ (homeostatic model assessment of insulin resistance) was calculated as fasting serum I (mU/L) multiplied by fasting plasma glucose (mmol/l) and divided by 22.1 [34]. Blood C-reactive protein (CRP) concentration was assessed by an immune test, using a diagnostic kit (CRPL2 Cobas Integra 400/800) from Roche.

### 2.6. Statistical Analysis

Age, body mass, body composition, and biochemical variables were expressed as mean ± SD. Before using the parametric test, the assumption of normality was verified using the Kolmogorov–Smirnov test. A one-way ANOVA was used with significance designated at *p* < 0.05. When appropriate, a Bonferroni post hoc test was used to compare selected data. Data analysis was performed with Statistica 12 software (StatSoft, Cracow, Poland).

## 3. Results

The subjects’ decision to join the U3A depended on their strong motivation to gain knowledge about pro-health behaviors. Dieting and nutrition courses (two semesters; 120 h) were provided by professionals from the Academy of Physical Education in Katowice. During this time, they also participated in physical exercise courses (Nordic walking, swimming, yoga, and gymnastics). After one year at the U3A, all participants were asked to prepare a 7 day dietary record to be analyzed in June. Daily diet compositions are presented in Table 2.

The U3A participants consumed a diet which resulted in significantly lower TEI (*p* < 0.05) than their TDEE (Table 1) (TEI: 1318.1 ± 327.4 kcal/d vs. TDEE: 2065.6 ± 169.3 kcal/d) and than the recommended dietary allowance (RDA) for women over 60 (1800–2100 kcal) [29]; they also had lower TEI than the CG (*p* < 0.05; Table 1). Significant differences in TEI (*p* < 0.05) and consumption of carbohydrates (*p* < 0.05), saccharose (*p* < 0.05), and protein (*p* < 0.05) were observed between the U3A group and the CG (Table 2). The U3A group consumed a significantly smaller amount of sodium (*p* < 0.05) compared with the CG (Table 2) and it was higher than RDA [29].

Table 2 presents the blood lipid profile and insulin resistance biomarkers measured in the U3A group and the CG. Subjects in the U3A group had a higher mean level of HDL-C (*p* < 0.05) and lower mean levels of TG (*p* < 0.05), I (*p* < 0.05), glucose (GL, *p* < 0.05), and HOMA-_IR_ (*p* < 0.05) than subjects in the CG (Table 3). Table 3 presents the participants’ body mass and compositions. Subjects from both groups had higher body masses and body fat percentages as well as greater VFAs, WCs, and BMIs.

Subjects in the U3A group performed at least 46.15 ± 5.12 min of PA per day with the intensity of 3–6 METs, which means moderate physical activity; this amounted to 323.05 ± 15.51 min per week. They had an average of 11,225 ± 156 steps/day. Subjects in the CG did not perform any PA per day and took an average of 2274 ± 325 steps/day. There were significant differences in TDEE between the groups (*p* < 0.05).

## 4. Discussion 

Our study included 106 women over 60 years old from the upper Silesia region of Poland. Sixty two of them were U3A students who volunteered for a one-year self-education program that included a course in nutrition (120 h). Forty four were not students of U3A and had no education in nutrition; this was the CG. In their nutrition course, the U3A group learned about the proper volume and frequency of meals and about the preferred foods healthy for individuals over the age of 60. Students were taught which food products were suitable sources of carbohydrates, fat, and protein and how often they should eat vegetables and fruits each day [35]. They also learned how to estimate their BMR and TDEE using the Harris–Benedict formula [29]. Our study was designed based on a cross-section methodology, and therefore the results were more prone to different biases, particularly the recall bias [36]. The results of this study suggest that despite the one-year nutritional education, subjects did not adhere to the healthy nutritional recommendations, which was indicated by the results of the 7 day nutritional analysis [6,37,38]. The data in both groups revealed several abnormalities in the U3A group with regard to the nutritional recommendations for this population [29]. There were no significant differences in the diet of the U3A group (who were well educated in nutrition) and the CG (who were not educated in nutrition). Subjects in the U3A group had an imbalance between their TEI and TDEE (Table 1) with an insufficient intake of basic macro and micronutrients [4,29]. Participants from the U3A group explained that despite the acquired knowledge, they were not able to apply it in their lives. Some of them attributed this to the high cost of healthy food and a lack of culinary skills [4,39]. Most of them explained that they lived alone and, therefore, had no motivation to cook for themselves or that they had so many other responsibilities that they had no time to go to the grocery shop or prepare meals [40,41]. Single females in the U3A group reported that, despite the knowledge they gained, they could not give up eating sweets or drinking small amounts of alcohol in the evenings as it made them feel happier, calmer, and more relaxed [40]. All of them unanimously stated that they were unaware that putting their acquired knowledge into practice would be so difficult [40]. Before they started attending the U3A, they thought that if they gained knowledge about a healthy diet and lifestyle they could easily apply it in practice [39]. As conversations with them showed, the main factors inhibiting healthier lifestyle changes were psychological rather than physiological [13]. Our results confirm that a change in eating behaviors requires not only knowledge of nutrition or cooperation with a dietitian, but also psychological care and motivation [13]. 

The diets of the elderly female students were not similar to the mixed diet recommended for this population [29] (Table 1). Diets of U3A students differed from diets of those in the CG, who did not participate in the nutrition course. It is worth mentioning that the diet of our subjects was still very similar to a typical diet of the Upper Silesia region. The golden rule of the Silesian dinner is a two course meal and a dessert. The U3A group decreased the size of the portions they consumed, but still chose unhealthy products, similar to those in the CG. A typical Silesian diet is as follows: for dinner, beef or pork meat rolled with bacon, pork chops, or pork stew is served. The meals are always served with Silesian potato dumplings, greased with fat. After dinner, coffee and a portion of cake (track pie, cheese cake with poppy seeds and jam) are served, too. For breakfast and supper, boiled or fried sausages and pates with bread are served. Vegetables are rare in the Silesian diet.

Our U3A subjects consumed slightly more calories than what they required for their BMR [6,29] and significantly less calories than their TDEE (*p* < 0.05) (Table 1). The high TDEE resulted from the high daily physical activity measured by the Actigraph during the seven days preceding the visit of the studied women to the laboratory to evaluate their body mass and composition, as well as biochemical variables. However, such a high level of physical activity of participants was not considered when the experiment was designed. They were not in the process of active weight reduction. It can be speculated that wearing the Actigraph may have been an incentive for women to perform additional physical activity, although this is only a speculation, not supported by any evidence. Unfortunately, we were not able to assess their physical activity in a more objective way but using the Actigraph [2,3,11]. The carbohydrate (*p* < 0.05), saccharose (*p* < 0.05), and protein (*p* < 0.05) consumption were significantly different between the U3A group and the CG. Lower saccharose consumption by the U3A group could influence their significantly lower glucose *(p* < 0.05), insulin (*p* < 0.05), and HOMA-_IR_ (*p* < 0.05) concentration. The U3A group consumed carbohydrates similar to the moderate carbohydrate diet [42]. The moderate carbohydrate diet (MCD) diet is recommended for patients with diabetes mellitus syndrome [6]. Most scientists confirm that carbohydrate-restricted diets improve serum HDL-C and reduce insulin and glucose concentration [42,43]. Abnormal concentrations of these variables are well-recognized risk factors for diabetes and cardiovascular diseases [43]. The U3A group consumed high amounts of fat, which contributed to more than 40% of their total calorie intake. Our participants consumed mainly proinflammatory saturated fatty acids (SFA), which can be associated with their high CRP level [12]. Predominant consumption of SFA causes an increase in tCh, LDL-C, and TG [12,44]. Our data are in agreement with previous studies conducted on a similar group of subjects that were characterized by a high level of body fat and an excessive BMI [45]. Furthermore, inadequate energy consumption and lower protein intake, 0.8 ± 0.01 g/kg.bm/d by women in the U3A group compared with 1–1.2 g protein/kg.bm/d for healthy elderly as recommended by the European Society for Clinical Nutrition and Metabolism, could influence their body composition [5,6,42,43]. Inadequate protein consumption can increase the reduction of lean body mass [43,44]. Liao et al. [6] reported that protein supplementation with PA, especially resistance training, promoted an increase in the muscle mass of older individuals. 

Loss of appetite and consequently lower daily energy intake is often observed among adults [29,45,46]. Eating inappropriate amounts of fiber, PUFA, and MUFA, or excess SFA intake promotes inflammation in the intestines and capillaries and blood lipid disorders. Unfortunately, very often people limit the consumption of valuable products such as meat, dairy products, and whole grain cereal products, but at the same time eat high-calorie snacks or sweets [12]. Xu et al. [28], while examining eating habits and obesity levels of Chinese adults, confirmed that a healthy balanced diet is essential in maintaining proper body mass and healthy aging. They analyzed data from 2745 elderly individuals using a 24 h recall over 3 consecutive days and concluded that modern dietary patterns included high intake of fruit, fast foods, and processed meat, what was associated with obesity. Similarly Shu et al. [47], in a cross-section study of 2560 individuals aged 45 to 60, revealed that in both genders animal food consumption was positively associated with the BMI and WC, whereas traditional Chinese diet rich in rice, pork, and vegetables was inversely associated with BMI and WC. A balanced diet should contain mainly complex sugars, whereas simple and disaccharide sugars should not exceed 10% of all sugars consumed. Excess consumption of simple sugars and inadequate fiber consumption is associated with the development of dyslipidemia, obesity, and insulin resistance. As Richards et al. [48] reported, high sugar and lower protein consumption may lead to insulin resistance and lower cognitive function in older community-dwelling adults. Furthermore, in U3A, inadequate fiber (10.5 g/1000 kcal vs. 14 g/1000 kcal recommended) and high sacharose and lactose (Table 1) consumption are other factors affecting the tCh and LDL-C concentrations as well as a high VFA [3]. In the elderly, lower fiber consumption can decrease intestinal peristalsis and increase digestive disorders [6]. In addition to macronutrients, a very important part of a healthy diet is correspondingly low salt intake. Excess salt in the diet is a factor strongly exacerbating hypertension, especially in people who are overweight and obese. The positive difference between the U3A group and the CG was that sodium was significantly lower (*p* < 0.05) in the U3A group’s diet [4,6]. Low sodium consumption can decrease blood pressure and prevent hypertension [49].

Inadequate protein consumption can decrease muscle mass and increase body fat stores [5,6,41,42]. Our subjects, in both groups, had very high VFA levels (Table 1) of almost 50 cm^2^ more than national recommendations (VFA > 100 cm^2^) [28]. The VFA is a factor that significantly influences the lipid profile [2]. Appropriate amounts of protein in the diet promote decreases in fat content [12]. It has been proven that meals rich in protein provide satiety for a longer period compared with those that are high in carbohydrates or fat, allowing to avoid snacks [41]. Additionally, the thermal effect of proteins (compared with carbohydrates or fats) promotes fat reduction in overweight or obese individuals [50]. 

A high level of the VFA also increases insulin resistance [2,50]. Despite having large amounts of body fat in the abdominal area, and consuming high amounts of fat (Table 1), participants in the U3A group presented an optimal level of fasting glucose and insulin and optimal HOMA-_IR_ [9,37]. The appropriate insulin and glucose levels were most likely associated with their relatively high level of daily PA [50] and low proportion of carbohydrates consumed in their diet (less than 50%) [12]. 

High body fat mass distribution, specifically the VFA, may result in metabolic disorders like atherosclerosis, hyperinsulinemia, and inflammation [46,51,52,53]. The VFA is correlated with cardiometabolic risk, even at a normal BMI, indicating the absence of obesity [54]. One of the inflammation markers is CRP [50]. In our study, we observed a high level of CRP in the blood (Table 2), confirming a moderate state of inflammation [46,50]. The high level of CRP in plasma and low vitamin C intake presented by our subjects could increase the generation of reactive oxygen species, which can promote the aging process [18,50,54]. The daily fat intake, with more than 50% coming from SFA in our participants, was excessive and reached a similar level as observed in Spanish [54] and French populations [55]. Another factor which can cause inflammation is inadequate consumption of vitamin D. Our subjects’ diets did not supply sufficient amounts of vitamin D (Table 1). It is known that vitamin D reduces proinflammatory cytokine production and increases anti-inflammatory cytokine production [53]. It was found that obese individuals had lower levels of vitamin D in the blood compared with subjects with a normal body mass [56]. The reduced Vitamin D levels in individuals with a higher amount of adipose tissue may be a consequence of vitamin D sequestration in adipose tissue [56]. In the elderly, the reduced ability to synthesize vitamin D and a lower sunlight exposure increase the importance of providing an appropriate amount of vitamin D through the diet [18]. 

PA is an invaluable factor for maintaining good health [22,57]. Regular, moderate exercise can reduce body fat content and improve the lipid profile and insulin resistance [13,30]. According to global health authorities, taking 10,000 steps per day, at a low or moderate intensity, is the minimum level of activity which improves physical and mental health [28]. Furthermore, the amount of time dedicated to PA, expressed in minutes, is important in preventing the development of civilization diseases [36]. Participating in 300 min of PA per week at moderate intensity is considered the minimum to maintain general health and fitness [56]. Our subjects performed at least 300 min of PA per week and averaged more than 10,000 steps per day. However, despite the high level of PA, our study subjects had abnormal levels of tCh and LDL-C and a larger VFA. Our results confirm that regular PA, without proper nutritional habits, is not a sufficient factor to maintain proper body mass and body composition and optimal lipoprotein levels [22]. For optimal health benefits, daily PA has to be supplemented with a well-balanced diet [57].

In summary, our study shows that the annual nutritional education did not help respondents to solve the problem of improper eating behavior. Changing eating behavior resulted mainly from mental rather than physiological limitations of study participants [40]. Our results revealed that despite the high motivation declared by the participants, they were not able to overcome their own mental weaknesses. This indicates that during courses, in addition to nutrition education and dietary care, students should also have psychological education and mental care [40]. To increase the effectiveness of dietary educational programs such as the one prescribed in this research, scientists should use additional measuring tools to evaluate eating behaviors, such as the semiquantitative food frequency questionnaire (FFQ) [47]. 

## 5. Limitations of the Study

Our study has some limitations, including a small sample size; the consideration of only female subjects; a lack of assessments across time, at least before and after the experiment. A further limitation of our study involved a lack of assessment of the levels of vitamins and minerals in the subjects’ blood. However, despite these limitations, we do believe that our findings may constitute a platform for further examination of the influence of diet on the general health of the elderly.

## 6. Conclusions

A healthy, balanced diet and an appropriate amount of daily PA are essential elements of healthy aging. Excess fat consumption results in obesity and other metabolic diseases, primarily lipid profile disorders and inflammation. Participants got enrolled in the courses at the U3A with a strong motivation to gain solid knowledge about proper nutrition and healthy behaviors. Compared with the CG who did not attend the one-year nutrition course, the U3A group consumed inappropriate amounts of calories and had an imbalanced diet regarding macronutrients. However, proper sodium consumption was observed in the U3A group. The U3A group also had imbalances in their body composition and a larger VFA. Despite the fact that the study population was well educated about a healthy balanced diet, they did not follow the basic principles of healthy eating in their everyday life.

## Figures and Tables

**Table 1:** Anthropometric characteristics of participants.

	U3A, n = 62 Mean ± SD	CG, n = 44 Mean ± SD
Height, cm	158.0 ± 4.8	159.3 ± 6.2
BM, kg	70.2 ± 13.3	72.3 ± 15.3
BMI	28.4 ± 1.2	28.5 ± 1.4
BMR, kcal	1291.1 ± 105.8	1310.2 ± 43.8
TDEE, kcal	2065.6 ± 169.3 *	1572 ± 52.6
BF %	38.9 ± 3.3	36.7 ± 6.5
VFA, cm^2^	146.0 ± 31.8	148.5 ± 21.8
WC, cm	94.1 ± 11.3	91.4 ± 12.8

* *p* < 0.05—statistically significant in relation to the CG, U3A—students from the University of the Third Age, CG—control group, BM—body mass, BMI—body mass index, BMR—basal metabolic rate, TDEE—total daily energy expenditure, BF—body fat, VFA—visceral fatty area, WC—waist circumference.

**Table 2:** Habitual energy intake, macronutrients, and vitamins and minerals consumption, as estimated from 7 day dietary records provided by participants.

Nutrients	U3A, n = 64 Mean ± SD	CG, n = 44 Mean ± SD	RDA
TEI, kcal/d	1318.1 ± 327.4 *	1553.25 ± 124.6	1800–2100
Macronutrients			
Carbs %	42	50	45–65
Carbs, g/d	138.4 ± 34.4 *	194.15 ± 15.2	nr
Carbs, g/kg.bm/d	2.0 ± 0.2	2.7 ± 0.2	nr
Saccharose, g/d	30.6 ± 12.8 *	50.8 ± 13.6	nr
Lactose, g/d	8.1 ± 2.1	11.3 ± 4.3	nr
Fiber, g/d	14.3 ± 7.4	17.72 ± 4.8	25
Fat %	41	32	20–35
Fat, g/d	60.0 ± 14.9	55.2 ± 8.4	43–76
Fat, g/kg.bm/d	1.2 ± 0.1	1.3 ± 0.5	nr
SFA, g/d	24.4 ± 8.8	26.3 ± 6.7	12–14
MUFA, g/d	23.2 ± 6.6	12.1 ± 4.6	nr
PUFA, g/d	12.4 ± 4.9	16.8 ± 6.3	nr
Cholesterol, g/d	199.2 ± 41.4	230 ± 32.7	300
Protein %	17	18	15–20
Protein, g/d	56.0 ± 13.9 *	69.9 ± 5.3	41–68
Protein, g/kg.bm/d	0.8 ± 0.1	0.96 ± 0.1	1
Vitamins			
Vit A, µg/d	588 ± 75.3	600 ± 45.8	770
Vit D, µg/d	4.0 ± 1.0	4.3 ± 1.3	15
Vit E, mg/d	7.0 ± 1.1	7.8 ± 2.1	10
Vit C, mg/d	38.8 ± 5.7	42.3 ± 8.3	85
Vit B_1_, mg/d	0.9 ± 0.1	1.1 ± 0.3	1.1
Vit B_2_, mg/d	1.2 ± 0.2	1.3 ± 0.3	1.1
Vit B_3_, mg/d	12 ± 2.2	13.1 ± 1.6	14
Vit B_6_, mg/d	1.4 ± 0.3	1.5 ± 0.7	1.5
Vit B_9_, μg/d	38.6 ± 5.2	45.2 ± 6.7	450
Vit B_12_, µg/d	4.0 ± 0.75	4.7 ± 1.4	2.8
Minerals			
Calcium, mg/d	459.5 ± 222.4	520.4 ± 127	1200
Sodium, mg/d	1560.2 ± 532*	3200 ± 230	1300
Potassium, mg/d	2400 ± 923.4	2542 ± 321	3500
Magnesium, mg/d	231.7 ± 102	285 ± 35.7	320
Iron, mg/d	8.4 ± 1.4	9.1 ± 2.4	10
Zinc, mg/d	7.6 ± 2.7	8.2 ± 1.8	6.8
Copper, mg/d	0.9 ± 0.08	1.1 ± 0.05	0.9

*—*p* < 0.05—statistically significant in relation to the CG, U3A—students from the University of the Third Age, CG—control group, Carbs—carbohydrates, SFA—saturated fatty acids, MUFA—mono unsaturated fatty acids, PUFA—poly unsaturated fatty acids, TEI—total energy intake, nr—no recommendations.

**Table 3:** The blood levels of lipid profiles and insulin resistance biomarkers of participants.

Variables	U3A, n = 62 Mean ± SD	CG, n = 44 Mean ± SD
TG, mg/dL	139.7 ± 45.3 *	179 ± 40.5
tCh, mg/dL	255.0 ± 49.5	246.5 ± 27.9
HDL-C, mg/dL	61.0 ± 19.1 *	45.3 ± 8.4
LDL-C, mg/dL	156.1 ± 53.8	172.6 ± 32.8
I, µg/dL	6.8 ± 3.1 *	9.8 ± 0.8
GL, mmol/L	5.8 ± 1.5 *	6.84 ± 0.4
HOMA-_IR_, mU/mmoL	1.75 ± 0.8 *	2.8 ± 0.5
CRP, mg/L	6.0 ± 1.7	5.5 ± 2.6

* *p* < 0.05—statistically significant in relation to the CG, U3A—students from the University of the Third Age, CG—control group, TG—triacylglycerol, tCh—total cholesterol, HDL-C—high-density lipoprotein cholesterol, LDL-C—low-density lipoprotein cholesterol, I—insulin, GL—glucose, HOMA-_IR_—the Homeostasis Model Assessment, CRP—C-reactive protein.

## References

[1] Murray S., Kroll C., Avedna N.M. (2015). Food and addiction among the aging population. Aging Res. Rev..

[2] Zając-Gawlak I., Kłapcińska B., Kroemeke A., Pośpiech D., Pelclová J., Přidalová M. (2017). Associations of visceral fat area and physical activity levels with the risk of metabolic syndrome in postmenopausal women. Biogerontology.

[3] Pelclová J., Štefelová N., Hodonská J., Dygrýn J., Gába A., Zając-Gawlak I. (2018). Reallocating Time from Sedentary Behavior to Light and Moderate-to-Vigorous Physical Activity: What Has a Stronger Association with Adiposity in Older Adult Women?. Int. J. Environ. Res. Public Health.

[4] Drewnowski A., Warren-Mears V.A. (2001). Does aging change nutrition requirements?. J. Nutr. Health Aging.

[5] Liao C.D., Tsauo J.Y., Wu Y.T., Cheng C.P., Chen H.C., Huang Y.C., Chen H.C., Liou T.H. (2017). Effects of protein supplementation combined with resistance exercise on body composition and physical function in older adults: A systematic review and meta-analysis. Am. J. Clin. Nutr..

[6] Skytte M.J., Samkani A., Petersen A.D., Thomsen M.N., Astrup A., Chabanova E., Frystyk J., Holst J.J., Thomsen H.S., Madsbad S. (2019). A carbohydrate-reduced high-protein diet improves HbA_1c_ and liver fat content in weight stable participants with type 2 diabetes: A randomised controlled trial. Diabetologia.

[7] Drewnowski A., Shultz J.M. (2001). Impact of aging on eating behaviors, food choices, nutrition, and health status. J. Nutr. Health Aging.

[8] Blumberg J. (1997). Nutritional needs of seniors. J. Am. Coll. Nutr..

[9] Lehtisalo J., Lindström J., Ngandu T., Kivipelto M., Ahtiluoto S., Ilanne-Parikka P., Keinänen-Kiukaanniemi S., Eriksson J.G., Uusitupa M., Tuomilehto J. (2016). Finnish Diabetes Prevention Study. Association of Long-Term Dietary Fat Intake, Exercise, and Weight with Later Cognitive Function in the Finnish Diabetes Prevention Study. J. Nutr. Health Aging.

[10] Zhou Q., Wu J., Tang J., Wang J.J., Lu C.H., Wang P.X. (2015). Beneficial Effect of Higher Dietary Fiber Intake on Plasma HDL-C and TC/HDL-C Ratio among Chinese Rural-to-Urban Migrant Workers. Int. J. Environ. Res. Public Health.

[11] Zając-Gawlak I., Pośpiech D., Kroemeke A., Mossakowska M., Gába A., Pelclová J., Přidalová M., Kłapcińska B. (2016). Physical activity, body composition and general health status of physically active students of the University of the Third Age (U3A). Arch. Gerontol. Geriatr..

[12] Michalczyk M., Zajac A., Mikolajec K., Zydek G., Langfort J. (2018). No Modification in Blood Lipoprotein Concentration but Changes in Body Composition after 4 Weeks of Low Carbohydrate Diet (LCD) Followed by 7 Days of Carbohydrate Loading in Basketball Players. J. Hum. Kinet..

[13] Hołowko J., Michalczyk M.M., Zając A., Czerwińska-Rogowska M., Ryterska K., Banaszczak M., Jakubczyk K., Stachowska E. (2019). Six Weeks of Calorie Restriction Improves Body Composition and Lipid Profile in Obese and Overweight Former Athletes. Nutrients.

[14] Friedrich M., Goluch-Koniuszy Z. (2015). Assessment of influence of pro-health nutrition education and resulting changes of nutrition behavior of women aged 65-85 on their body content. Prz. Menopauzalny.

[15] Wakimoto P., Block G. (2001). Dietary intake, dietary patterns, and changes with age: An epidemiological perspective. J. Gerontol. A Biol. Sci. Med. Sci..

[16] Ritz P. (2001). Factors affecting energy and macronutrient requirements in elderly people. Public Health Nutr..

[17] Fluitman K.S., Nadar H.J., Roos D.S., Berends H.W., Keijser B.F.J., Nieuwdorp M., Ijzerman R.G., Visser M. (2019). The association of olfactory function with BMI, appetite and prospective weight change in Dutch community- dwelling older adults. J. Nutr. Health Aging.

[18] Elmadfa I., Meyer A.L. (2008). Body composition, changing physiological functions and nutrient requirements of the elderly. Ann. Nutr. Metab..

[19] Vincent D., Lauque S., Lanzmann D., Vellas B., Albarede J.L. (1998). Changes in dietary intakes with age. J. Nutr. Health Aging.

[20] Marshal C., Lengyel C. (2012). Body dissatisfaction among middle-age and older women. Can. J. Diet. Pract. Res..

[21] Mangweth-Matzek B., Hoek H., Pope H.G. (2014). Pathological eating and body dissatisfaction in middle-age and older women. Curr. Opin. Psychiatry.

[22] Pelclová J., Gába A., Tlučáková L., Pośpiech D. (2012). Association between physical activity (PA) guidelines and body composition variables in middle-aged and older women. Arch. Gerontol. Geriatr..

[23] Booth F.W., Roberts C.K., Laye M.J. (2012). Lack of exercise is a major cause of chronic diseases. Compr. Physiol..

[24] Cho B.C.K., Pak A.W.P., Choi J.C.L., Choi E.C.L. (2007). Daily step goal of 10,000 steps: A literature review. Clin. Investig. Med. Médecine Clin. Exp..

[25] Garber C.E., Blissmer B., Deschenes M.R., Franklin B.A., Lamonte M.J., Lee I.M., Nieman D.C., Swain D.P. (2011). American College of Sports Medicine position stand. Quantity and quality of exercise for developing and maintaining cardiorespiratory, musculoskeletal, and neuromotor fitness in apparently healthy adults: Guidance for prescribing exercise. Med. Sci. Sports Exerc..

[26] Xu X., Hall J., Byles J., Shi Z. (2015). Dietary Pattern Is Associated with Obesity in Older People in China: Data from China Health and Nutrition Survey (CHNS). Nutrients.

[27] Friedrich M. (2004). Health-promoting education in nutrition as a factor modifying dietary habits in menopausal women. Przegl. Lek..

[28] Friedrich M. (2005). Effects of health-promoting nutritional education and change in dietary habits on visceral fatty tissue contents and on concentration of insulin and cortosol in menopausal women. Pol. J. Food Nutr. Sci..

[29] Jarosz M. (2017). Standards of nutrition for the Polish population—Amendment [in polish]. Inst. Food Nutr..

[30] Herrmann S.D., Barreira T.V., Kang M., Ainsworth B.E. (2013). How many hours are enough? Accelerometer wear time may provide bias in daily activity estimates. J. Phys. Act. Health.

[31] Esliger D.W., Copeland J.L., Barnes J.D., Tremblay M.S. (2005). Standardizing and optimizing the use of accelerometer data for free-living physical activity monitoring. J. Phys. Act. Health.

[32] Ogawa H., Fujitani K., Tsujinaka T., Imanishi K., Shirakata H., Kantani A., Hirao M., Kurokawa Y., Utsumi S. (2011). InBody 720 as a new method of evaluating visceral obesity. Hepatogastroenterology.

[33] Friedewald W.T., Levy R.I., Fredrickson D.S. (1972). Estimation of the concentration of low-density lipoprotein cholesterol in plasma, without use of the preparative ultracentrifuge. Clin. Chem..

[34] Wallace T.M., Matthews D.R. (2002). The assessment of insulin resistance in man. Diabet. Med..

[35] Schnebelen-Berthier C., Negro N., Jaruga A., Roques C.F., Lecerf J.M. (2019). Long term effect of spa therapy combined with patient education program on subjects with overweight and obesity—A controlled study. Obes. Res. Clin. Pract..

[36] Pilis K., Pilis A., Stec K., Pilis W., Langfort J., Letkiewicz S., Michalski C., Czuba M., Zych M., Chalimoniuk M. (2018). Three-Year Chronic Consumption of Low-Carbohydrate Diet Impairs Exercise Performance and Has a Small Unfavorable Effect on Lipid Profile in Middle-Aged Men. Nutrients..

[37] Maciejewska D., Michalczyk M., Czerwińska-Rogowska M., Banaszczak M., Ryterska K., Jakubczyk K., Piotrwski J., Hołowko J., Drozd A., Wysokińki P. (2017). Seeking Optimal Nutrition for Healthy Body Mass Reduction among Former Athletes. J. Hum. Kinet..

[38] Lam M.C.L., Adams J. (2017). Association between home food preparation skills and behaviour, and consumption of ultra-processed foods: Cross-sectional analysis of the UK National Diet and nutrition survey (2008–2009). Int J Behav Nutr Phys Act.

[39] Wolfson J.A., Bleich S.N. (2015). Is cooking at home associated with better diet quality or weight-loss intention?. Public Health Nutr..

[40] Phan U.T., Chambers E. (2016). Motivations for choosing various food groups based on individual foods. Appetite.

[41] Kirk J.K., Graves D.E., Craven T.E., Lipkin E.W., Austin M., Margolis K.L. (2008). Restricted-carbohydrate diets in patients with type 2 diabetes: A meta-analysis. J. Am. Diet. Assoc..

[42] Accurso A., Bernstein R.K., Dahlqvist A., Draznin B., Feinman R.D., Fine E.J., Gleed A., Jacobs D.B., Larson G., Lustig R.H. (2008). Dietary carbohydrate restriction in type 2 diabetes mellitus and metabolic syndrome: Time for a critical appraisal. Nutr. Metab. (Lond)..

[43] Deutz N.E., Bauer J.M., Barazzoni R., Biolo G., Boirie Y., Bosy-Westphal A., Cederholm T., Cruz-Jentoft A., Krznariç Z., Nair K.S. (2014). Protein intake and exercise for optimal muscle function with aging: Recommendations from the ESPEN Expert Group. Clin. Nutr..

[44] Westman E.C., Vernon M.C. (2008). Has carbohydrate-restriction been forgotten as a treatment for diabetes mellitus? A perspective on the ACCORD study design. Nutr. Metab. (Lond)..

[45] Sturm K., MacIntosh C.G., Parker B.A., Wishart J., Horowitz M., Chapman I.M. (2003). Appetite, food intake, and plasma concentrations of cholecystokinin, ghrelin, and other gastrointestinal hormones in undernourished older women and well-nourished young and older women. J. Clin. Endocrinol. Metab..

[46] Wright A.J., Southon S., Bailey A.L., Finglas P.M., Maisey S., Fulcher R.A. (1995). Nutrient intake and biochemical status of non-instutionalized elderly subjects in Norwich: Comparison with younger adults and adolescents from the same general community. Br. J. Nutr..

[47] Shu L., Zheng P.F., Zhang X.Y., Si C.J., Yu X.L., Gao W., Zhang L., Liao D. (2015). Association between Dietary Patterns and the Indicators of Obesity among Chinese: A Cross-Sectional Study. Nutrients.

[48] Richard E.L., Laughlin G.A., Kritz-Silverstein D., Reas E.T., Barrett-Connor E., McEvoy L.K. (2018). Dietary Patterns and Cognitive Function among Older Community-Dwelling Adults. Nutrients.

[49] Ozemek C., Laddu D.R., Arena R., Lavie C.J. (2018). The role of diet for prevention and management of hypertension. Curr. Opin. Cardiol..

[50] Anderson-Vasquez H.E., Pérez-Martínez P., Ortega F.P., Wanden-Berghe C. (2015). Impact of the consumption of a rich diet in butter and it replacement for a rich diet in extra virgin olive oil on anthropometric, metabolic and lipid profile in postmenopausal women. Nutr. Hosp..

[51] Racette S.B., Evans E.M., Weiss E.P., Hagberg J.M., Holloszy J.O. (2006). Abdominal adiposity is a stronger predictor of insulin resistance than fitness among 50–95 year olds. Diabetes Care.

[52] Ortega R.M., Requejo A.M., Andrés P., López-Sobaler A.M., Redondo R., González-Fernández M. (1995). Relationship between diet composition and body mass index in a group of Spanish adolescents. Br J Nutr.

[53] Fontana L., Villareal D.T., Weiss E.P., Racette S.B., Steger-May K., Klein S., Holloszy J.O. (2007). Calorie restriction or exercise: Effects on coronary heart disease risk factors. A randomized, controlled trial. Am. J. Physiol..

[54] Ortega R.M., Requejo A.M., Andrés P., López-Sobaler A.M., Quintas M.E., Redondo M.R., Navia B., Rivas T. (1997). Dietary intake and cognitive function in a group of elderly people. Am. J. Clin. Nutr..

[55] Kesse- Guyot E., Andreeva V.A., Jeandel C., Ferry M., Hercberg S., Galan P. (2012). A healthy dietary pattern at midlife is associated with subsequent cognitive performance. J. Nutr..

[56] Salehpour A., Hosseinpanah F., Shidfar F., Vafa M., Razaghi M., Dehghani S., Hoshiarrad A., Gohari M. (2012). A 12-week double-blind randomized clinical trial of vitamin D₃ supplementation on body fat mass in healthy overweight and obese women. Nutr. J..

[57] Wilkinson R., Marmot M.G. (2003). Social Determinations of Health: The Solid Facts.

